# Supplementary material for: Multi‐dataset identification of innovative feature genes and molecular mechanisms in keratoconus
Source: J Cell Mol Med. 2024 Sep 19;28(18):e70079. doi: 10.1111/jcmm.70079 (PMC11412914; doi:10.1111/jcmm.70079)
Supplement: Supplementary file 2 — Material S2. [file JCMM-28-e70079-s002.zip › 7.GSEA analysis/2.ATOH7/KEGG_NEUROTROPHIN_SIGNALING_PATHWAY.html]

Details for gene set KEGG\_NEUROTROPHIN\_SIGNALING\_PATHWAY[GSEA]

|  || Dataset | ATOH7.group.cls#h\_versus\_l |
| Phenotype | group.cls#h\_versus\_l |
| Upregulated in class | h |
| GeneSet | KEGG\_NEUROTROPHIN\_SIGNALING\_PATHWAY |
| Enrichment Score (ES) | 0.28657857 |
| Normalized Enrichment Score (NES) | 1.3726758 |
| Nominal p-value | 0.103515625 |
| FDR q-value | 0.33165017 |
| FWER p-Value | 0.925 |
Table: GSEA Results Summary

  

Fig 1: Enrichment plot: KEGG\_NEUROTROPHIN\_SIGNALING\_PATHWAY      
 Profile of the Running ES Score & Positions of GeneSet Members on the Rank Ordered List

  

| SYMBOL | TITLE | RANK IN GENE LIST | RANK METRIC SCORE | RUNNING ES | CORE ENRICHMENT || 1 | AKT3 | na | 9 | 1.043 | 0.0754 | Yes |
| 2 | IRS1 | na | 55 | 0.848 | 0.1356 | Yes |
| 3 | SHC2 | na | 290 | 0.628 | 0.1743 | Yes |
| 4 | BCL2 | na | 1098 | 0.432 | 0.1823 | Yes |
| 5 | NTRK2 | na | 1392 | 0.398 | 0.2027 | Yes |
| 6 | CAMK2D | na | 2442 | 0.313 | 0.1951 | Yes |
| 7 | CALML6 | na | 3497 | 0.256 | 0.1832 | Yes |
| 8 | IRS2 | na | 3662 | 0.249 | 0.1966 | Yes |
| 9 | SH2B3 | na | 4005 | 0.236 | 0.2038 | Yes |
| 10 | MAP2K1 | na | 4240 | 0.228 | 0.2135 | Yes |
| 11 | YWHAH | na | 4637 | 0.213 | 0.2175 | Yes |
| 12 | CAMK4 | na | 4659 | 0.212 | 0.2323 | Yes |
| 13 | NFKBIE | na | 5212 | 0.194 | 0.2305 | Yes |
| 14 | RPS6KA1 | na | 5757 | 0.178 | 0.2276 | Yes |
| 15 | CRKL | na | 5867 | 0.175 | 0.2372 | Yes |
| 16 | PIK3R3 | na | 6166 | 0.166 | 0.2406 | Yes |
| 17 | RELA | na | 6248 | 0.164 | 0.2501 | Yes |
| 18 | MAPK12 | na | 6613 | 0.154 | 0.2507 | Yes |
| 19 | NTF3 | na | 7205 | 0.138 | 0.2437 | Yes |
| 20 | NTF4 | na | 7333 | 0.135 | 0.2498 | Yes |
| 21 | RIPK2 | na | 7407 | 0.133 | 0.2573 | Yes |
| 22 | RAPGEF1 | na | 7417 | 0.132 | 0.2666 | Yes |
| 23 | MAP3K5 | na | 7445 | 0.132 | 0.2754 | Yes |
| 24 | NGF | na | 7651 | 0.127 | 0.2787 | Yes |
| 25 | NRAS | na | 7693 | 0.126 | 0.2866 | Yes |
| 26 | NFKB1 | na | 8680 | 0.105 | 0.2657 | No |
| 27 | CRK | na | 8997 | 0.099 | 0.2637 | No |
| 28 | MAGED1 | na | 9326 | 0.093 | 0.2610 | No |
| 29 | MAPKAPK2 | na | 9943 | 0.082 | 0.2492 | No |
| 30 | CALM3 | na | 10070 | 0.080 | 0.2514 | No |
| 31 | IRAK2 | na | 10201 | 0.078 | 0.2533 | No |
| 32 | GRB2 | na | 10441 | 0.075 | 0.2518 | No |
| 33 | NFKBIA | na | 10503 | 0.073 | 0.2553 | No |
| 34 | SHC4 | na | 10680 | 0.071 | 0.2554 | No |
| 35 | RPS6KA6 | na | 10771 | 0.070 | 0.2578 | No |
| 36 | SH2B1 | na | 11147 | 0.064 | 0.2516 | No |
| 37 | IRAK4 | na | 11468 | 0.059 | 0.2467 | No |
| 38 | SH2B2 | na | 11701 | 0.056 | 0.2441 | No |
| 39 | FOXO3 | na | 12029 | 0.052 | 0.2384 | No |
| 40 | YWHAQ | na | 12136 | 0.051 | 0.2390 | No |
| 41 | ARHGDIA | na | 12453 | 0.047 | 0.2333 | No |
| 42 | BDNF | na | 12595 | 0.045 | 0.2325 | No |
| 43 | NFKBIB | na | 13350 | 0.037 | 0.2133 | No |
| 44 | TRAF6 | na | 13633 | 0.034 | 0.2077 | No |
| 45 | MAPK1 | na | 14536 | 0.025 | 0.1834 | No |
| 46 | HRAS | na | 14559 | 0.025 | 0.1846 | No |
| 47 | TP53 | na | 14698 | 0.024 | 0.1824 | No |
| 48 | SHC1 | na | 15160 | 0.020 | 0.1705 | No |
| 49 | RAF1 | na | 15219 | 0.019 | 0.1702 | No |
| 50 | MAP2K5 | na | 15356 | 0.018 | 0.1676 | No |
| 51 | RHOA | na | 15382 | 0.018 | 0.1681 | No |
| 52 | AKT1 | na | 15787 | 0.014 | 0.1575 | No |
| 53 | GSK3B | na | 16028 | 0.013 | 0.1515 | No |
| 54 | PIK3CD | na | 16202 | 0.011 | 0.1473 | No |
| 55 | YWHAG | na | 16222 | 0.011 | 0.1476 | No |
| 56 | CALM1 | na | 16251 | 0.011 | 0.1475 | No |
| 57 | RAC1 | na | 16331 | 0.010 | 0.1460 | No |
| 58 | ABL1 | na | 16492 | 0.009 | 0.1420 | No |
| 59 | JUN | na | 16587 | 0.008 | 0.1399 | No |
| 60 | MAPK3 | na | 16821 | 0.006 | 0.1336 | No |
| 61 | RAP1A | na | 16926 | 0.005 | 0.1309 | No |
| 62 | MAPK8 | na | 17140 | 0.003 | 0.1250 | No |
| 63 | BAD | na | 17218 | 0.002 | 0.1230 | No |
| 64 | ATF4 | na | 17294 | 0.002 | 0.1209 | No |
| 65 | BAX | na | 17508 | 0.000 | 0.1148 | No |
| 66 | MAPK7 | na | 17787 | -0.002 | 0.1069 | No |
| 67 | CDC42 | na | 17874 | -0.003 | 0.1046 | No |
| 68 | PRKCD | na | 17924 | -0.003 | 0.1035 | No |
| 69 | RAP1B | na | 18052 | -0.005 | 0.1001 | No |
| 70 | MAP2K7 | na | 18710 | -0.010 | 0.0819 | No |
| 71 | MAP3K1 | na | 18900 | -0.012 | 0.0773 | No |
| 72 | GAB1 | na | 19058 | -0.013 | 0.0737 | No |
| 73 | SORT1 | na | 19079 | -0.014 | 0.0741 | No |
| 74 | FRS2 | na | 19349 | -0.016 | 0.0675 | No |
| 75 | ARHGDIB | na | 19726 | -0.019 | 0.0580 | No |
| 76 | YWHAB | na | 19781 | -0.020 | 0.0579 | No |
| 77 | YWHAZ | na | 20490 | -0.027 | 0.0394 | No |
| 78 | CALML3 | na | 20596 | -0.028 | 0.0383 | No |
| 79 | MAP2K2 | na | 20653 | -0.028 | 0.0387 | No |
| 80 | CSK | na | 20669 | -0.028 | 0.0404 | No |
| 81 | PIK3CB | na | 20685 | -0.028 | 0.0420 | No |
| 82 | RPS6KA3 | na | 20863 | -0.030 | 0.0391 | No |
| 83 | TP73 | na | 20879 | -0.030 | 0.0408 | No |
| 84 | MAPK10 | na | 20989 | -0.032 | 0.0400 | No |
| 85 | MAP3K3 | na | 21084 | -0.032 | 0.0396 | No |
| 86 | PIK3CA | na | 21359 | -0.036 | 0.0343 | No |
| 87 | KIDINS220 | na | 21690 | -0.039 | 0.0276 | No |
| 88 | CALM2 | na | 21949 | -0.042 | 0.0232 | No |
| 89 | PIK3R2 | na | 22209 | -0.045 | 0.0190 | No |
| 90 | CAMK2A | na | 22272 | -0.046 | 0.0205 | No |
| 91 | SOS2 | na | 22550 | -0.049 | 0.0161 | No |
| 92 | ZNF274 | na | 22575 | -0.049 | 0.0189 | No |
| 93 | RPS6KA4 | na | 22603 | -0.050 | 0.0218 | No |
| 94 | PTPN11 | na | 22613 | -0.050 | 0.0251 | No |
| 95 | PRDM4 | na | 22614 | -0.050 | 0.0287 | No |
| 96 | SHC3 | na | 22782 | -0.052 | 0.0276 | No |
| 97 | PSEN1 | na | 22990 | -0.055 | 0.0256 | No |
| 98 | CAMK2G | na | 23214 | -0.058 | 0.0234 | No |
| 99 | IRS4 | na | 23255 | -0.058 | 0.0264 | No |
| 100 | MAPK14 | na | 23324 | -0.059 | 0.0288 | No |
| 101 | IKBKB | na | 23356 | -0.060 | 0.0322 | No |
| 102 | MAPK13 | na | 23983 | -0.069 | 0.0191 | No |
| 103 | NTRK3 | na | 24155 | -0.072 | 0.0194 | No |
| 104 | PDPK1 | na | 24308 | -0.074 | 0.0204 | No |
| 105 | PLCG2 | na | 24577 | -0.078 | 0.0183 | No |
| 106 | MAPK9 | na | 24826 | -0.082 | 0.0171 | No |
| 107 | IRAK1 | na | 24885 | -0.084 | 0.0215 | No |
| 108 | BRAF | na | 25048 | -0.086 | 0.0231 | No |
| 109 | KRAS | na | 25083 | -0.087 | 0.0284 | No |
| 110 | PLCG1 | na | 25238 | -0.090 | 0.0304 | No |
| 111 | NTRK1 | na | 25343 | -0.091 | 0.0340 | No |
| 112 | MAPK11 | na | 25479 | -0.094 | 0.0369 | No |
| 113 | YWHAE | na | 25594 | -0.096 | 0.0406 | No |
| 114 | PIK3CG | na | 26210 | -0.109 | 0.0307 | No |
| 115 | RPS6KA5 | na | 26273 | -0.110 | 0.0369 | No |
| 116 | IRAK3 | na | 26437 | -0.113 | 0.0404 | No |
| 117 | SOS1 | na | 26852 | -0.122 | 0.0373 | No |
| 118 | AKT2 | na | 27111 | -0.128 | 0.0391 | No |
| 119 | RPS6KA2 | na | 27234 | -0.132 | 0.0452 | No |
| 120 | PIK3R1 | na | 27720 | -0.144 | 0.0415 | No |
| 121 | FASLG | na | 29443 | -0.193 | 0.0058 | No |
| 122 | CALML5 | na | 29931 | -0.208 | 0.0068 | No |
| 123 | NGFR | na | 30175 | -0.217 | 0.0155 | No |
| 124 | PIK3R5 | na | 34383 | -0.611 | -0.0618 | No |
| 125 | CAMK2B | na | 34693 | -0.989 | 0.0010 | No |
Table: GSEA details [plain text format]

  

Fig 2: KEGG\_NEUROTROPHIN\_SIGNALING\_PATHWAY      
 Blue-Pink O' Gram in the Space of the Analyzed GeneSet

  

Fig 3: KEGG\_NEUROTROPHIN\_SIGNALING\_PATHWAY: Random ES distribution      
 Gene set null distribution of ES for **KEGG\_NEUROTROPHIN\_SIGNALING\_PATHWAY**
